# Sociodemographic Determinants of Nonattendance in a Population-Based Mammography Screening Program in the City of Manisa, Turkey

**DOI:** 10.1100/2012/816903

**Published:** 2012-03-12

**Authors:** Pınar Erbay Dundar, Beyhan Cengiz Ozyurt, Koray Erdurak

**Affiliations:** ^1^Department of Public Health, Faculty of Medicine, Celal Bayar University, İstasyon Mevkii, 45020 Manisa, Turkey; ^2^Public Health Specialist, Cancer Control Unit, Manisa Health Directorate, Akmescit Mah, İzmir Caddesi No. 33, 45010 Manisa, Turkey

## Abstract

*Objectives*. Community based breast cancer screening has decreased breast cancer mortality in women. This study examined the predictors of nonattendence for invitational breast cancer screening in relation to socioeconomic status in the city of Manisa, in western Turkey. *Study Design*. For the evaluation of the reasons for refusing to participate in the study, two districts were selected. 446 women aged between 50 and 69 years were selected from the program database by systematic random sampling. *Methods*. The questionnaire consisted of sociodemographic variables and the adapted version of Champion's Health Belief Model Scale. Univariete and multivariete logistic regression analysis were performed throughout the data analysis. *Results*. Being from an urban district and being from the western region were the risk factors for not participating in the screening program (*P* = 0.014, *P* = 0.023). A statistical significance was found between mammography-benefit, mammography-barrier and program participation (*P* = 0.044, *P* = 0.006). Although there were many more barriers for not participating in the screening program for the women of the slum district, the attendence rate of the slum district was higher than that of the urban district. *Conclusions*. Increased attendance may be achieved through enhancement of breast cancer awareness and by reducing some of the modifiable barriers.

## 1. Introduction

Breast cancer (BC) is the most common cancer among women worldwide. It is the most prevalent cause of cancer-related death in women in both developed and developing countries [1]. According to the data of the Ministry of Health, breast cancer incidence in Turkey is 35.8 per hundred thousand [2]. 10065 new breast cancer cases and 4311 deaths associated with breast cancer were reported in 2008 [3]. While the 5-year-survival rate of patients diagnosed with breast cancer for all phases is 73% in developed countries, it is reported to be 53% in developing countries. The significant difference between these figures can be explained by the difference of early diagnosis and better treatment capabilities, owing to screening mammography. Several studies have indicated that community-based breast cancer screening (clinical breast examination-CBE plus mammography), executed for an appropriate age range at appropriate intervals and with quality assurance in every step, has decreased breast cancer mortality in women by 30%. International organisations have differing recommendations concerning the age group that should receive mammography and at what frequency; the most appropriate is that every country should develop standards according to its own conditions. The standard developed by the Cancer Control Department of the Ministry of Health for Turkey advises that women of the 50–69 age group have bilateral mammography every two years [4]. Cancer Early Diagnosis and Screening Centers (KETEM—Kanser Erken Teshis, Tarama ve Eğitim Merkezleri) have been established in order to execute population-based screening programs for cancers recommended by the WHO for screening. By the end of the year 2008, the ministry had established on its own 84 centers in 81 provinces. The duties of KETEMs are to organize training that aims to inform health personnel and the public about the issues of cancer and to raise awareness; to provide diagnosis at early stages through population based screening programs of identified risk groups in line with established screening standards (for breast, cervical, colorectal cancers, etc.); to refer the patients diagnosed with cancer to treatment centers with the necessary medical guidance; to carry out patient followup and evaluations and to provide as much social, psychological and medical support as possible. In Turkey population-based mammography screening was introduced in 2006. Routine biennial CBE and mammography screening are free of charge in the KETEMs. High rates of participation among the target population are needed to achieve the reductions in mortality evidenced by clinical trials and organized programs [5, 6]. But this goal has not been reached yet. Opportunistic or self-referral mammography frequency varies from 10.7 to 34.0% in Turkey [7–11].

Acceptance of mammography may be related to sociodemographic characteristics such as age, level of education, or social class [12, 13] or to health service utilization patterns, specifically doctor visits in the previous year, physician recommendation of mammography, and access to a regular source of health care [14–17]. Adherence to other preventive practices, benign breast disease, and a family history of breast cancer are also factors often associated with the decision to undergo mammography [14–16]. One of the most important factors that influence women's decisions about whether to undergo mammography is the nature of their health beliefs. If a woman perceives the benefits of mammography to be high and barriers to mammography to be low, she is more likely to undergo regular mammography. In order to identify predictors of nonattendance for invitational breast cancer screening in relation to socioeconomic status, we conducted a cross-sectional study among women identified in a mammography register in the city of Manisa, in western Turkey. The specific research aims were to (a) describe the sociodemographic characteristics, knowledge, beliefs, and mammography screening practices of 50–69-year-old women and (b) identify which of the above factors are predictive of mammography screening.

## 2. Methods

### 2.1. Study Population

The study was conducted among women invited to participate in the breast cancer early detection program in the Manisa KETEM. The nurses working in the KETEM invited the women to participate in the study by visiting them at their houses or telephoning them. A protocol was made with the municipality to supply means of transport. The women were taken by car from health centers close to their homes to the KETEM building and were taken back after the mammography had been performed. Driving women to their appointments was a standard approach in 2008-2009 when the study was being performed in Manisa KETEM. However different approaches are used in some other provinces such as invitation only via mail. For the evaluation of the reasons for refusing to participate in the study, regarding the social status and health beliefs, two regions in the Manisa city center were selected. The Uncubozköy district (urban) is inhabited by educated women of western origin and the Mevlana district (slum) is where uneducated women, who migrated from eastern cities, and their commonly unemployed husbands live. In general, women from the western region of Turkey, are less traditional and better educated than those in the general population of Turkey. The attitudes of the husbands and the religious beliefs are not barriers for women's health practices in western Turkey. Since other regions, especially the eastern part of Turkey are more traditional than the western region, migration may be an important marker for attitudes.

The study population were women aged between 50 and 69 years invited to undergo a mammography during the second year of the first round of the breast cancer screening program (2008-2009) in these two districts. Women who had had a mammogram during the previous year, had been diagnosed with breast cancer, had a mental or physical handicap, or had an unknown address were excluded from the study.

The study population consisted of two districts with different socioeconomic characteristics. Women from each district were selected from the program database by systematic random sampling. The total population of the two regions was 892 women aged between 50 and 69 years. Half of the total population (*n* = 446) was defined as the sample size. The participation rate of the study was 81.2% (*n* = 362). When a power calculation was looked at retrospectively, power of the study was determined 85% in *d* = 0.20 and *n* = 360. Ten trained intern doctors from the medical faculty collected the data in face-to-face interviews between April and May 2010. They made clear the confidentiality, benefits, risks, and future implications of the research. Data were then collected from those who had verbally consented to participate.

For the women who were absent, the visits were repeated once. And if they were not found on the second visit either, these women were excluded from the study. Interviews were done at the women's houses and each lasted an average of 30 minutes.

The study was ethically approved by the Manisa Province Health Directorate.

### 2.2. Variables

The questionnaire consisted of sociodemographic variables, a form regarding risk factors and signs of breast cancer, and the measurement of the health belief model of breast cancer. Sociodemographic measures, including characteristics such as the respondent's age, current marital status, level of education, income level, family type, and migration state, were assessed. The perceived income level was recorded as a marker for the determination of the economic level, and it was coded as sufficient = 1 or insufficient = 2.

The subjects were also asked if they had any knowledge about breast cancer and if there were family members and/or friends with breast cancer histories. 18 questions were used to determine the individuals' level of knowledge of breast cancer. The answers were “true = 1,” “false = 0,” and “do not know = 0.” The knowledge score was computed by totalling the number of correct answers for all 18 questions. The knowledge score was recoded into dichotomous variables by taking the mean value as the cutoff value to evaluate knowledge levels, coded sufficient = 1 and insufficient = 2.

Champion's Health Belief Model Scale (CHBMS) was also applied to the subjects. The Health Belief Model Scale was developed in 1984 and was revised in later works by Champion [18, 19]. It was adapted into Turkish, validated, and tested for reliability in several studies [10, 20, 21]. The adaptations of Gözüm and Aydin and the mammography subscales were used in this study [10]. A total of 33 items are in the scale categorized as follows: susceptibility (3 items), seriousness (7 items), health motivation (7 items), benefits-mammography (5 items), and barriers-mammography (11 items). All the items have 5 response choices ranging from strong disagreement (1 point) to strong agreement (5 points). All scales are positively related to screening behaviour, except for barriers which are negatively associated. A high score therefore meant that the subject believed she had greater susceptibility to breast cancer, perceived breast cancer risk to be more serious, but also perceived increased benefits and fewer barriers, had more confidence in both breast self-examination and mammography, and in general had higher health motivation [18]. All subscales were positively related to screening behaviors except barriers, which were scored inversely. The subjects were also asked about reasons for nonattendance at the screening.

### 2.3. Statistical Analysis

We computed odds ratios (ORs) and 95% confidence intervals (CIs) using the SPSS v10.0 statistical package. Chi-square test was applied in categorical variables. To examine the effects of the independent variables on the odds of being a nonattender, we conducted a univariate logistic regression analysis. All items were treated as categorical variables in the analysis. In a second step, only the subscales significant in the univariate analyses were tested in a multivariate model. Student's *t* test was also used in comparisons of continuous variables.

## 3. Results

The women's mean age in the slum district was 58.3 ± 5.7. 93.2% of women were illiterate, 87.9% came from eastern Turkey, 62.1% had an insufficient income level, and 98.5% were housewives. Nearly half of the husbands were unemployed. 

85.5% of women in an urban district were literate, 83.3% came from western Turkey, 84.6% had a sufficient income level, and 76.3% were housewives. Statistically significant differences were found according to districts and sociodemographic features (Table 1). 

47.2% of the study group reported that they had heard or read about breast cancer. 51.4% had sufficient knowledge of it. Health professionals were mentioned as the main source of information on BC by 52.6% of the participants (data not shown). 

The sociodemographic characteristics of the study group according to participation in mammography screening are summarized in Table 2. Being from an urban district and being from the western region were the statistically significant factors for not participating in the screening program. Family history of breast cancer and level of knowledge about BC were not associated with participation in the program. A statistical significance was found between mammography-benefit, mammography-barrier, and program participation (*P* = 0.044, *P* = 0.006). The mean score of mammography-benefit was lower, and the mean score of mammography-barrier was higher in the nonattending group. Susceptibility, seriousness, and motivation were not significant variables in program participation. 

All women provided a reason for not participating. Among the reasons (Table 3), “other health problems” for the slum district and “already having had mammography somewhere else” for the urban district were the most frequent. Failure to receive an invitation (phone call or visiting) was also often mentioned. 

Benefit and barrier subscales were significant variables in program participation. When a univariate analysis was performed, one benefit and eight barrier items were significant in relation to nonattendance (Table 4). In the logistic regression analysis, one benefit and three barrier items were significant. Women who disagreed that “mammography detects lumps before they can be felt” were nearly five times more likely to be nonattenders. In the barrier subscale, women who considered that mammography is painful were more than two times more likely to be nonattenders. Furthermore, women who were dissatisfied with health personnel were 4.8 times and women who thought that they were not old enough for periodic mammography screening were three times more likely to be nonattenders (Table 5). To examine the effects of the district on the odds of being a nonattender, univariate and multivariate logistic regression analysis was performed. The item “mammography is painful” was the risk factor for nonattendance in the urban district. The items “mammography reduces the risk of dying from BC,” “mammography makes me worry about having BC,” and “I am dissatisfied with the health care personnel” were the risk factors for not participating in the screening program in the slum district (Table 6). 

## 4. Discussion 

In this study, participation in the screening program was 76.8%. 97.1% of the attenders stated that they were satisfied, and 84.9% of them declared future intentions to obtain a mammogram. This attendance rate was consistent with other community-based screening of western cities in Turkey [22]. According to a study carried out in Israel, mammography rates were approximately 20% [23]. In the USA, mammography rates were found to range from 48.5% to 74.5% in recent years [24–26]. In this program, after being invited by KETEM nurses who visited them at their homes or telephoned them in person, the women who accepted to participate were taken to the KETEM building and back by car. Also, in the slum, the call from the local authority proved effective. The high attendance rate is due to these factors/facilities. Women from the urban district were less likely to participate in the screening program than those of the slum district. However, it should be recognized that a greater proportion of the urban women had had a mammogram in a public/private clinic. Previous studies have suggested that opportunistic programs or self-referral mammographies attract women with medium-to-high levels of education [14, 16, 27], whereas organized programs tend to attract women from lower social classes [13, 15, 28, 29]. However, other studies have not reported education-related differences in participation [28, 30–34]. 

In this study, the sociodemographic variables and the perceived income level were not significant in relation to attendance. Similarly, in a study by Lagerlund it was concluded that sociodemographic factors alone do not appear to constitute strong predictors of nonattendance [34]. In general, older women are more reluctant to undergo mammography. These women tend to have a lower perception of their breast cancer risk and display more negative attitudes towards screening [12]. At the same time, older women receive less frequent physician recommendations for mammography [35]. This association has predominantly been reported by studies in areas that lack organized programs [12, 36, 37]. However, differences in participation among the different age groups are reduced in population-based programs, consistent with the results of our study [12, 29, 33, 34, 38, 39]. Recruitment methods used by organized programs, as well as efforts to ensure equal access for all eligible women, may foster equal access for all age groups. In a study which described inequalities in the use of breast and cervical cancer screening services according to socioeconomic position and educational level in European countries, inequalities are higher in countries without population-based cancer screening programmes. These results highlight the potential benefits of population-based screening programmes [40]. 

Our main findings are that barriers and benefits represented the major determinants of participation in mammography. Health beliefs are important in the process of stimulating positive health behaviors in specific populations. Women who undergo regular mammograms report fewer barriers and perceive more benefits from the screening process [12, 41]. Nonparticipation is more common among women with greater emotional barriers or those who fear that mammography will be painful [28, 34]. Some authors [28, 30] have reported higher participation rates among women with higher knowledge of the usefulness of mammography. In our study, two dimensions of the scale were predictive of attendance. The mean score of the benefit was lower and the mean score of the barrier was higher in the nonattending group. Our finding that the perceived barriers represented the most prominent predictor of nonattendance corroborates the results of most previous studies but not all [42]. Elimination of barriers would seem to be an attractive way to increase attendance. For example, the belief that “I am not old enough for mammography screening periodically” or “mammography is painful” could be changed by information. But, to find the reason of discontent against health workers, qualitative studies are needed. 

Knowledge about both the lifetime risk of breast cancer [43] and the treatment options [44] has been shown to be related to the attendance at mammography screening. But in this study, a family history of breast cancer or the level of knowledge about BC is not related to participation in the program, as previous studies have reported [14, 34]. This was because these women were informed about breast cancer in the KETEM before screening. This homogeny of the knowledge level among the women may be the reason for the lack of a significant difference relating to these factors. 

Advice, recommendation, or encouragement from health professionals hase been found to increase the likelihood of attendance in most previous studies [45–47]. According to experiences in the United States, advice from the medical profession to have a mammogram is a leading determinant of attendance. This finding is important for this study also. The persistent invitations and advice provided by both the screening center and the family doctors seem to be the most important reasons of the high attendance rates. In addition, especially in the slum district, the participation of the local authority in the program is a good example for the official advice and advocacy increasing the attendance. In this district where the traditional lifestyle reigns, the local authority, by talking to the husbands and persuading them about the usefulness of breast cancer screening, played a pivotal role in encouraging attendance. There are several studies showing relations between religious thoughts and perceived seriousness, disclosing that Muslim women tend to avoid screenings because of their fatalistic beliefs [48, 49]. In this study, no relationship between nonattendance and perceived seriousness and susceptibility was found. When multivariate logistic regression analysis was performed to examine the effects of the district on the odds of being a nonattender, it was found that the item “mammography is painful” was the single risk factor for nonattendance in the urban district. Although there were many more barriers for not participating in the screening program for the women of the slum district, the attendence rate of the slum district was higher than that of the urban district. 

There are several limitations in this study. Potential weaknesses include its retrospective design. It is problematic question of whether attitudes influence behavior or whether a mammography experience influences one's attitudes and knowledge. The smaller sample size in this study may have limited to detect any effect of the independent variables such as age and level of education. 

In conclusion, our results indicate that increased attendance may be achieved through enhancement of breast cancer awareness and by reducing some of the modifiable barriers. 

Suggested interventions include previous contacts with the health care system, better information before screening, a friendly screening atmosphere and empathetic, supportive staff behaviour, encouraging them to feel more at ease and distracted from pain. Elimination of barriers would seem to be an attractive way to increase attendance.

The persistent invitations and the advice provided by both the screening center and the family doctors, as well as the participation of the local authority in advocacy, were of crucial importance, especially in women from socioeconomically disadvantaged regions. 

## Figures and Tables

**Table 1:** Sociodemographic characteristics and knowledge levels about BC of women according to districts.

	Urban (*n* = 156)	Slum (*n* = 206)	*P* value*
*Age* (mean ± sd)	57.1 ± 5.9	59.0 ± 5.5	0.002**
*Marital status*	%	%	
Single	1.3	0.5	
Currently married	82.7	60.7	0.000
Widowed/separated	16.0	38.8	
*Working activity*			
Employed-retired	23.7	1.5	0.000
Housewife	76.3	98.5	
Husband's job^§^			
Unskilled blue collar	7.8	10.3	
Skilled blue collar	22.5	23.0	
Unskilled white collar	38.0	4.0	
Skilled white collar	12.4	1.6	0.000
Self employed	11.6	12.7	
Unemployed	7.8	48.4	
*Educational level*			
Illiterate-incomplete primary	14.5	93.2	
Primary	50.6	6.8	0.000
Secondary/above	34.9	0.0	
*Place of birth *			
Eastern region	16.7	87.9	0.000
Western region	83.3	12.1	
*Perceived family income*			
Sufficient	84.6	37.9	0.000
Insufficient	15.4	62.1	
*Knowledge level about BC*			
Sufficient	76.9	32.0	0.000
Insufficient	23.1	68.0	

**^§^** *n* = 129 for urban, 126 for slum.

*Chi-square test.

**Student's *t* test.

**Table 2:** Sociodemographic characteristics, knowledge level, family history of BC, and health beliefs of attenders and nonattenders to mammography screening program.

Variables	Nonattenders, *n*(%)	Attenders, *n*(%)	*P* value*
	(*n* = 84)	(*n* = 278)	
*Region*			
Urban	46 (29.5)	110 (70.5)	0.014
Slum	38 (18.4)	168 (81.6)	
*Age *			
50–59	53 (24.2)	166 (75.8)	0.578
60–69	31 (21.7)	112 (78.8)	
*Education level*			
İlliterate	43 (20.1)	171 (79.9)	0.092
Primary/above	41 (27.7)	107 (72.3)	
*Place of birth *			
Western region	45 (29.0)	110 (71.0)	0.023
Eastern region	39 (18.8)	168 (81.2)	
*Perceived family income*			
Sufficient	55 (26.2)	155 (73.8)	0.114
Insufficient	29 (19.1)	123 (80.9)	
*Knowledge level on BC*			
Sufficient	49 (26.3)	137 (73.7)	0.146
Insufficient	35 (19.9)	141 (80.1)	
*Family history of BC*			
Present	7 (30.4)	16 (69.6)	0.396
Absent	77 (22.7)	262 (77.3)	
Health belief model scale*	mean ± sd	mean ± sd	
Susceptibility	7.0 ± 2.2	7.1 ± 2.4	0.549
Seriousness	21.8 ± 4.4	21.7 ± 4.3	0.843
Motivation	18.7 ± 3.6	19.0 ± 3.0	0.521
MMG benefit	18.0 ± 3.3	18.8 ± 2.8	0.044
MMG barrier	28.1 ± 7.7	25.5 ± 6.2	0.006

*Chi-square test

**Student's *t* test.

**Table 3:** Principal reasons stated for nonattendance to mammography screening program.

Cause of nonattendance	Slum, (*n* = 38)	Urban, (*n* = 46)
I did not receive the invitation	7	10
No need for screening	1	1
I was at work	1	7
I already have had MMG in public/private clinic	5	20
I was afraid of having BC	3	3
I was afraid from radiation	—	1
I was embarrassed	3	—
MMG is painful	3	1
Other health problems	15	3

**Table 4:** Univariate ORs and 95% CIs of nonattendance at mammography screening for single-item variables that were statistically significant in a univariate analysis.

Variables	Nonattenders	Attenders	OR (95%CI)
*Benefits*			
*Mammography detects lumps before they can be felt*			
agree	55 (19.5)	227 (80.5)	1.0
undecided	23 (33.3)	46 (66.7)	2.1 (1.2–3.7)
disagree	6 (54.5)	5 (45.5)	4.9 (1.5–16.8)
*Barriers*			
*Mammography makes me worry about having BC*			
disagree	53 (19.8)	215 (80.2)	1.0
undecided	8 (36.4)	14 (63.9)	2.3 (0.9–5.8)
agree	23 (31.9)	49 (68.1)	1.9 (1.1–3.4)
*I do not know the procedure*			
disagree	59 (20.1)	234 (79.9)	1.0
undecided	9 (36.0)	16 (64.0)	2.2 (0.9–5.3)
agree	16 (36.4)	28 (63.6)	2.3 (1.2–4.5)
*The mammographic examination is troublesome*			
disagree	45 (18.4)	199 (81.6)	1.0
undecided	23 (48.9)	24 (51.1)	4.2 (2.2–8.2)
agree	16 (22.5)	55 (77.5)	1.3 (0.7–2.4)
*Hard to find time to go for a mammographic examination*			
disagree	57 (18.3)	223 (81.7)	1.0
undecided	28 (41.8)	39 (58.2)	3.2 (1.8–5.7)
agree	6 (27.3)	16 (72.7)	1.7 (0.6–4.5)
*Mammography is painful*			
disagree	33 (16.8)	163 (83.2)	1.0
undecided	30 (48.4)	32 (51.6)	4.6 (2.5–8.6)
agree	21 (20.2)	83 (79.8)	1.3 (0.7–2.3)
*Discontent with health care personnel*			
disagree	53 (17.4)	252 (82.6)	1.0
undecided	29 (59.2)	20 (40.8)	6.9 (3.6–13.1)
agree	2 (25.0)	6 (75.0)	1.5 (0.3–8.1)
*Have too many other problems*			
disagree	37 (18.7)	161 (81.3)	1.0
undecided	12 (22.6)	41 (77.4)	1.3 (0.6–2.7)
agree	35 (31.5)	76 (68.5)	2.0 (1.2–3.4)
*I am not old enough for mammography screening periodically*			
disagree	50 (19.4)	208(80.6)	1.0
undecided	15 (22.1)	53 (77.9)	1.2 (0.6–2.3)
agree	19 (52.8)	17 (47.2)	4.7 (2.3–9.6)

Agree 1-2, undecided 3, disagree 4-5 on Likert scale for benefits

Agree 4-5, undecided 3, disagree 1-2 on Likert scale for barriers.

**Table 5:** Multivariate OR's of nonattendance at mammography screening.

Variable	Multivariate OR (95%)
*Mammography detects lumps before they can be felt*	
(disagree)	4.8 (1.2–18.2)
*Mammography is painful*	
(undecided)	2.6 (1.2–6.0)
*Discontent with health care personnel *	
(undecided)	4.8 (2.0–11.4)
*I am not old enough for mammography screening periodically*	
(agree)	3.3 (1.5–7.5)

**Table 6:** Multivariate OR's of nonattendance at mammography according to districts.

Variable	Urban multivariate OR (95%)	Slum multivariate OR (95%)
*Mammography is painful*		
(undecided)	4.3 (1.0–18.2)	
*Mammography reduces the risk of dying BC*		
(disagree)		6.4 (1.4–30.3)
*Mammography makes me worry about having BC*		
(agree)		9.2 (1.9–43.1)
*Discontent with health care personnel*		
(undecided)		11.5 (3.4–33.4)

## Appendices

### A. Questionnaire of Sociodemographic Determinants of Mammography

#### A.1. Sociodemographic Variables

1. Age —
2. Education level
  1. illiterate
  2. literate
  3. primary school
  4. secondary school
  5. high school
  6. university graduate

(3) Women's job
  1. housewife
  2. employed
  3. retired

(4) Marital status
  1. married
  2. single
  3. widow
  4. separated

((5), (6), (7), and (8) questions are for married women)

(5) Husband's job
  1. unskilled blue collar
  2. skilled blue collar
  3. unskilled white collar
  4. skilled white collar
  5. self employed
  6. unemployed

(6) Husband's education level
  1. illiterate
  2. literate
  3. primary school
  4. secondary school
  5. high school
  6. university graduate

(7) Husband's age —

(8) Family type
  1. core
  2. expanded
  3. separated

(9) Perceived family income level
  1. sufficient
  2. insufficient

(10) History of migration
  1. yes
  2. no

(11) If the answer is yes which region?
  1. Western Anatolia
  2. Central Anatolia
  3. Marmara Region
  4. Northern Anatolia
  5. Eastern Anatolia
  6. Southeastern Anatolia
  7. Southern Anatolia

#### A.2. Risk Factors of Breast Cancer

(12) Menarche age —

Questions For Married

(13) parity
  1. yes
  2. no
(14) age at first delivery —
(15) breastfeeding
  1. yes
  2. no
(16) history of menopause
  1. yes
  2. no
(17) smoking
  1. yes (— day)
  2. never
  3. ex-smoker
(18) Information about breast cancer? (if “yes” source of information)
  1. no information
  2. health professionals
  3. books/brochures/magazines
  4. friends-neighborhood
  5. TV-radio
  6. other
(19) Family history of breast cancer (your mother, sister, aunt, grandmother)
  1. no
  2. yes

#### A.3. Questions about Knowledge Level of Breast Cancer

(20) What is the effect of aging on breast cancer probability?
  1. increase
  2. decrease
  3. no effect
  4. do not know

(21) What is the effect of nulliparity on breast cancer probability?
  1. increase
  2. decrease
  3. no effect
  4. do not know
(22) What is the effect on breast cancer probability if first delivery age is above 30?
  1. increase
  2. decrease
  3. no effect
  4. do not know
(23) What is the effect on breast cancer probability if menopause age is above 50?
  1. increase
  2. decrease
  3. no effect
  4. do not know
(24) What is the effect on breast cancer probability if menarche age is under 11?
  1. increase
  2. decrease
  3. no effect
  4. do not know
(25) What is the probability of counter-lateral cancer formation in breast cancer patients?
  1. increase
  2. decrease
  3. no effect
  4. do not know
(26) What is the effect on breast cancer probability if family history is present?
  1. increase
  2. decrease
  3. no effect
  4. do not know
(27) What is the effect of obesity on breast cancer probability?
  1. increase
  2. decrease
  3. no effect
  4. do not know
(28) What is the effect of oral contraceptives on breast cancer probability?
  1. increase
  2. decrease
  3. no effect
  4. do not know
(29) What is the effect of breastfeeding on breast cancer probability?
  1. increase
  2. decrease
  3. no effect
  4. do not know
(30) What is the effect of using alcohol on breast cancer probability?
  1. increase
  2. decrease
  3. no effect
  4. do not know
(31) What is the effect of smoking on breast cancer probability?
  1. increase
  2. decrease
  3. no effect
  4. do not know
(32) What is the effect of radiation exposure on breast cancer probability?
  1. increase
  2. decrease
  3. no effect
  4. do not know
(33) What is the effect of having benign breast disease on breast cancer probability?
  1. increase
  2. decrease
  3. no effect
  4. do not know
(34) What is the effect of hormone replacement therapy on breast cancer probability?
  1. increase
  2. decrease
  3. no effect
  4. do not know
(35) Do you know breast self-examination?
  1. I do not know
  2. Yes I know, but have never applied
  3. I apply whenever it comes to my mind
  4. Once in a month

(36) Do you know what mammography is?
  1. I do not know
  2. yes I know, but never underwent
  3. once in a year
  4. every two years
(37) What can be done for early diagnosis in breast cancer?
  1. breast self-examination
  2. clinical breast examination
  3. mammography
  4. do not know
  5. there is no early diagnosis procedure for breast cancer

(38) Do you have any information about mammography screening by conducting KETEM?
  1. yes
  2. no
(39) Have you attended mammography screening program by conducting KETEM?
  1. yes
  2. no (skip to the 41st question)

(40) If you attended mammography screening program, are you satisfied?
  1. yes
  2. no
(41) Principal reasons stated for nonattendance to mammography screening program
  1. I did not receive the invitation
  2. no need for screening
  3. I was at work
  4. I already have had mammography in public/private clinic
  5. I was afraid of having breast cancer
  6. I was afraid from radiation
  7. I was embarrassed
  8. Mammography is painful
  9. Other health problems
(42) Will you attend the next mammography screening program?
  1. yes
  2. no

### B. Champion's Revised Health Belief Model Scales

*Scale Items*

#### B.1. Susceptibility

1. My chances of getting breast cancer are great.
  ( ) strong disagreement
  ( ) disagreement
  ( ) undecided
  ( ) agreement
  ( ) strong agreement
2. There is a good possibility I will get breast cancer in the next years.
  ( ) strong disagreement
  ( ) disagreement
  ( ) undecided
  ( ) agreement
  ( ) strong agreement
3. I feel I will get breast cancer in the future.
  ( ) strong disagreement
  ( ) disagreement
  ( ) undecided
  ( ) agreement
  ( ) strong agreement

#### B.2. Seriousness

1. The thought of breast cancer scares me.
  ( ) strong disagreement
  ( ) disagreement
  ( ) undecided
  ( ) agreement
  ( ) strong agreement
2. When I think about breast cancer, my heart beats faster.
  ( ) strong disagreement
  ( ) disagreement
  ( ) undecided
  ( ) agreement
  ( ) strong agreement
3. I am afraid to think about breast cancer.
  ( ) strong disagreement
  ( ) disagreement
  ( ) undecided
  ( ) agreement
  ( ) strong agreement
4. Problems I would experience with breast cancer would last a long time.
  ( ) strong disagreement
  ( ) disagreement
  ( ) undecided
  ( ) agreement
  ( ) strong agreement
5. Breast cancer would threaten a relationship with my boyfriend, husband, or partner.
  ( ) strong disagreement
  ( ) disagreement
  ( ) undecided
  ( ) agreement
  ( ) strong agreement
6. If I had breast cancer my whole life would change.
  ( ) strong disagreement
  ( ) disagreement
  ( ) undecided
  ( ) agreement
  ( ) strong agreement
7. If I developed breast cancer, I would not live longer than 5 years.
  ( ) strong disagreement
  ( ) disagreement
  ( ) undecided
  ( ) agreement
  ( ) strong agreement

#### B.3. Health Motivation

1. I want to discover health problems early.
  ( ) strong disagreement
  ( ) disagreement
  ( ) undecided
  ( ) agreement
  ( ) strong agreement
2. Maintaining good health is extremely important to me.
  ( ) strong disagreement
  ( ) disagreement
  ( ) undecided
  ( ) agreement
  ( ) strong agreement
3. I search for new information to improve my health.
  ( ) strong disagreement
  ( ) disagreement
  ( ) undecided
  ( ) agreement
  ( ) strong agreement
4. I feel it is important to carry out activities which will improve my health.
  ( ) strong disagreement
  ( ) disagreement
  ( ) undecided
  ( ) agreement
  ( ) strong agreement
5. I eat well-balanced meals.
  ( ) strong disagreement
  ( ) disagreement
  ( ) undecided
  ( ) agreement
  ( ) strong agreement
6. I exercise at least 3 times a week.
  ( ) strong disagreement
  ( ) disagreement
  ( ) undecided
  ( ) agreement
  ( ) strong agreement
7. I have regular health check-ups even when I am not sick.
  ( ) strong disagreement
  ( ) disagreement
  ( ) undecided
  ( ) agreement
  ( ) strong agreement

#### B.4. Benefits-Mammogram

1. When I get a recommended mammogram, I feel good about myself.
  ( ) strong disagreement
  ( ) disagreement
  ( ) undecided
  ( ) agreement
  ( ) strong agreement
2. When I get a mammogram, I do not worry as much about breast cancer.
  ( ) strong disagreement
  ( ) disagreement
  ( ) undecided
  ( ) agreement
  ( ) strong agreement
3. Having a mammogram of the breast will help me find lumps early.
  ( ) strong disagreement
  ( ) disagreement
  ( ) undecided
  ( ) agreement
  ( ) strong agreement
4. Having a mammogram of the breast will decrease my chances of dying from breast cancer.
  ( ) strong disagreement
  ( ) disagreement
  ( ) undecided
  ( ) agreement
  ( ) strong agreement
5. Having a mammogram will help me find a lump before it can be felt by myself or a health professional.
  ( ) strong disagreement
  ( ) disagreement
  ( ) undecided
  ( ) agreement
  ( ) strong agreement

#### B.5. Barriers-Mammogram

1. Having a routine mammogram of the breast would make me worry about breast cancer.
  ( ) strong disagreement
  ( ) disagreement
  ( ) undecided
  ( ) agreement
  ( ) strong agreement
2. I fear having a mammogram because I do not know the procedure
  ( ) strong disagreement
  ( ) disagreement
  ( ) undecided
  ( ) agreement
  ( ) strong agreement
3. I do not know where and how to have the mammogram.
  ( ) strong disagreement
  ( ) disagreement
  ( ) undecided
  ( ) agreement
  ( ) strong agreement
4. Having a mammogram of the breast would be troublesome.
  ( ) strong disagreement
  ( ) disagreement
  ( ) undecided
  ( ) agreement
  ( ) strong agreement
5. Having a mammogram of the breast would take too much time.
  ( ) strong disagreement
  ( ) disagreement
  ( ) undecided
  ( ) agreement
  ( ) strong agreement
6. Having a mammogram of the breast would be painful.
  ( ) strong disagreement
  ( ) disagreement
  ( ) undecided
  ( ) agreement
  ( ) strong agreement
7. I am discontent with health care personnel, they behave rude.
  ( ) strong disagreement
  ( ) disagreement
  ( ) undecided
  ( ) agreement
  ( ) strong agreement
8. Having a mammogram of the breast would expose me to unnecessary radiation.
  ( ) strong disagreement
  ( ) disagreement
  ( ) undecided
  ( ) agreement
  ( ) strong agreement
9. I cannot remember to consult having a mammogram.
  ( ) strong disagreement
  ( ) disagreement
  ( ) undecided
  ( ) agreement
  ( ) strong agreement
10. I have too many other problems than having a mammogram.
  ( ) strong disagreement
  ( ) disagreement
  ( ) undecided
  ( ) agreement
  ( ) strong agreement
11. I am not old enough for mammography screening periodically.
  ( ) strong disagreement
  ( ) disagreement
  ( ) undecided
  ( ) agreement
  ( ) strong agreement
